# SNP-IT Tool for Identifying Subspecies and Associated Lineages of *Mycobacterium tuberculosis* Complex 

**DOI:** 10.3201/eid2503.180894

**Published:** 2019-03

**Authors:** Samuel Lipworth, Rana Jajou, Albert de Neeling, Phelim Bradley, Wim van der Hoek, Gugu Maphalala, Maryline Bonnet, Elizabeth Sanchez-Padilla, Roland Diel, Stefan Niemann, Zamin Iqbal, Grace Smith, Tim Peto, Derrick Crook, Timothy Walker, Dick van Soolingen

**Affiliations:** University of Oxford, Oxford, UK (S. Lipworth, T. Peto, D. Crook, T. Walker);; National Institute for Public Health and the Environment, Bilthoven, the Netherlands (R. Jajou, A. de Neeling, W. van der Hoek, D. van Soolingen);; Wellcome Trust Centre for Human Genetics, Oxford (P. Bradley);; National Reference Laboratory, Ministry of Health, Mbabane, Swaziland (G. Maphalala);; Epicentre, Paris, France (M. Bonnet, E. Sanchez-Padilla); University of Kiel, Kiel, Germany (R. Diel);; Borstel Research Centre, Borstel, Germany (S. Niemann);; European Bioinformatics Institute, Cambridge, UK (Z. Iqbal);; Public Health England, Birmingham, UK (G. Smith)

**Keywords:** tuberculosis, whole-genome sequencing, zoonoses, phylogeny, single-nucleotide polymorphism, SNP-IT, Mycobacterium tuberculosis, bacteria, United Kingdom, TB

## Abstract

The clinical phenotype of zoonotic tuberculosis and its contribution to the global burden of disease are poorly understood and probably underestimated. This shortcoming is partly because of the inability of currently available laboratory and in silico tools to accurately identify all subspecies of the *Mycobacterium tuberculosis* complex (MTBC). We present SNPs to Identify TB (SNP-IT), a single-nucleotide polymorphism–based tool to identify all members of MTBC, including animal clades. By applying SNP-IT to a collection of clinical genomes from a UK reference laboratory, we detected an unexpectedly high number of *M. orygis* isolates. *M. orygis* is seen at a similar rate to *M. bovis*, yet *M. orygis* cases have not been previously described in the United Kingdom. From an international perspective, it is possible that *M. orygis* is an underestimated zoonosis. Accurate identification will enable study of the clinical phenotype, host range, and transmission mechanisms of all subspecies of MTBC in greater detail.

*Mycobacterium tuberculosis* complex (MTBC) encompasses a group of organisms that cause tuberculosis (TB) in humans and animals. TB in humans is caused mainly by *M. tuberculosis* but also by other members of MTBC, including the less well understood animal-associated subspecies *M. bovis*, *M. caprae*, *M. pinnipedii*, *M. suricattae*, *M. orygis*, *M. microti*, and *M. mungi* (*1**–**6*). The global burden of zoonotic TB is thought to be both underestimated and increasing (*7*); however, accurate assessment of prevalence is made difficult by a lack of clinical diagnostic tools and surveillance (*8*).

Efforts to differentiate members of MTBC and study the phylogeny of the complex have thus far included analysis of large genomic deletions (*9*), variable-number tandem-repeats (VNTR), spacer oligonucleotide typing (spoligotyping), multilocus sequence typing, and, more recently, single-nucleotide polymorphism (SNP)–based phylogenies (*10*). Numerous tools now exist that make in silico predictions of lineages within the complex from whole-genome sequencing (WGS) data using a variety of approaches, including the detection of single SNPs from both unassembled and mapped genomes, comparison of de Brujin graphs, and MinHash-based comparisons (*11*–*14*). None of these tools has yet been calibrated to reliably differentiate among all subspecies, particularly the animal-associated ones, whose incidence and clinical significance are likely to be underestimated as a result.

The host ranges of the various MTBC subspecies differ, which has serious implications for contact investigations and source case finding. For example, *M. microti* is found in wild cats and rodents and causes human infection, usually in association with rodent contact (*15*). In contrast with infections caused by *M. bovis,* most *M. microti* infections have been reported to cause pulmonary TB, which raises the possibility of onward transmission, although such transmission has not yet been reported (*16*). *M. pinnipedii*, which causes TB in seals, is sometimes transmitted to humans during outbreaks in zoos or wildlife parks (*17*). Although isolated mostly from gazelle species, *M. orygis* has also been seen in humans in recent years, although how humans contract this bacterium is still unclear (*2*). *M. mungi* causes disease in banded mongooses, the dassie bacillus causes disease in rock hyraxes, and *M. suricattae* causes disease in meerkats, but none of these bacteria is currently known to cause disease in humans (*3*,*5*).

The spectrum of clinical phenotypes associated with human infection by MTBC animal lineages is largely unknown, partly because the identification of these organisms is currently difficult. Accurate identification of the causative subspecies in all cases would enable characterization of disease associated with animal lineages and of diversity in clinical phenotypes, which would contribute to better disease management. A higher level of knowledge on the spread and host range of the subspecies would also provide a better basis on which to study the history of the evolutionary development of the complex as a whole. Now that routine WGS is being performed by Public Health England (PHE) for all MTBC isolates, we sought to use these data to estimate the burden of animal-associated TB in England. Therefore, we identified a broad panel of SNPs that define each subspecies, lineage, and sublineage within the MTBC and assessed them using a new SNP-based tool, SNPs to Identify TB (SNP-IT).

## Materials and Methods

### Calibration Set

**Figure 1:**
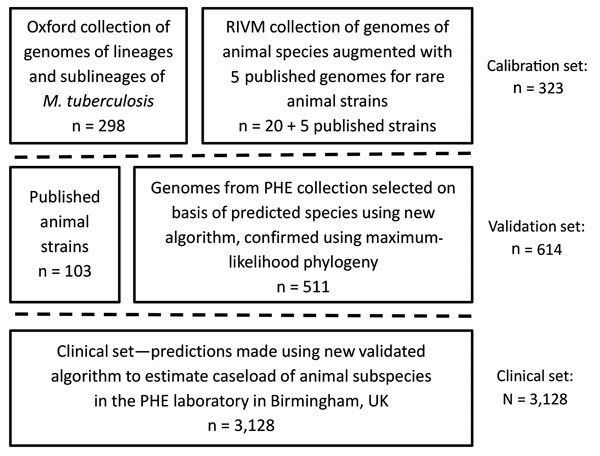
Description of the *Mycobacterium tuberculosis* complex datasets used in the 3 stages of calibration, validation, and application to a clinical set of the new SNPs to Identify TB tool. PHE, Public Health England (Birmingham, UK); RIVM, Netherlands National Institute for Public Health and the Environment (Bilthoven, the Netherlands); SNP, single-nucleotide polymorphism.

We defined a set of isolates (N = 323) from which to identify SNPs associated with subspecies, lineages, and sublineages within the MTBC (Figure 1). We identified isolates from the collection of the National Institute for Public Health and the Environment (RIVM; Bilthoven, Netherlands) using a combination of spoligotyping patterns, SNPs, restriction fragment length polymorphism (RFLP) patterns, the hybridization patterns in the HAIN Genotype MTBC assay, polymorphic GC-rich repeat sequence (PGRS) profiles, and VNTR patterns in accordance with current and previous standard practice (Appendix Table 1). We identified isolates from a whole-genome sequencing archive held at the University of Oxford (Oxford, UK) using the SNP typing system of Stucki et al. (*18*) for Mtb lineages 1–4 and by clustering on a maximum likelihood tree with the isolates from the Netherlands for lineages 5 and 6. In addition, we used published strains (n = 5) to be able to include *M. suricattae, M. mungi*, and the dassie bacillus, which were not present in the Oxford or RIVM collections. The nomenclature we adopted for this study is summarized in Appendix Table 2.

### Bioinformatics

We applied parallel bioinformatics approaches to assess applicability across pipelines. As such, we independently mapped reads from Illumina platforms (https://www.illumina.com) to 2 different versions of the H37Rv reference genome. We mapped reads to NC000962.3 with Breseq version 0.28.1 (http://barricklab.org/twiki/bin/view/Lab/ToolsBacterialGenomeResequencing), using a minimum allele frequency of 80% and minimum coverage of 5 times for SNP calls. Separately, we mapped reads again to NC000962.2, for which we used Snippy version 3.1 with default settings (minimum coverage 10 times, minimum allele frequency 90%) (*19*,*20*). We extracted all SNPs shared exclusively by isolates of each subspecies, lineage, and sublineage identified by both pipelines. The lineage-defining positions for lineage 4 are not variants with respect to the reference, itself lineage 4, but are uniquely conserved positions. We therefore identified these positions by mapping a core SNP alignment to a maximum-likelihood tree using Mesquite version 3.30 (*21*). These nucleotide loci were added to the catalog of phylogeny-determining SNPs.

All newly sequenced genomes are available from the National Center for Biotechnology Information under project accession no. PRJNA418900. The SNP-IT tool, including all relevant reference libraries used for this study, is available as an open-source package online (https://github.com/samlipworth/snpit).

### Validation Set

To validate the algorithm, we compiled an independent collection of genomes (N = 511) using clinical isolates sequenced by PHE Birmingham, UK, identified as MTBC and not included in the calibration set (Figure 1). We augmented them with data from the European Nucleotide Archive and the National Center for Biotechnology Information Sequence Read Archive to increase the representation of animal subspecies (N = 103; Appendix Table 2). To maximize inclusion of animal isolates from the public archives, we used the new Colored Bloom Graph (CBG) software (*22*). Using CBG, we searched a snapshot of the Sequence Read Archive (to December 2016, N = 455,632) with our new set of reference kmers for Mykrobe predictor (see Comparison with Existing Tools).

We compared FASTA files of the whole genome (https://www.ebi.ac.uk/Tools/sss/fasta) to the catalog of phylogeny-determining SNPs to make predictions for PHE isolates, whereas for isolates downloaded from the nucleotide archive, we created only limited variant calling format files using Snippy (only SNPs with respect to the reference genome were included to increase computational efficiency). To ensure that genomic loci defining lineage 4 were included, we used a mutated reference genome to create these limited variant calling format files. We compared SNPs in the query sample with reference libraries of lineage-specific SNPs for each clade. We assigned query genomes to particular subspecies or lineages if >10% of lineage- or subspecies-specific SNPs were detected in the strain in question. We assessed all predictions against the maximum-likelihood phylogeny. For *M. mungi*, we could locate only 1 genome in the public sequence libraries, so we could not validate this subspecies.

### Clinical Isolates

To assess the caseload across the different members of the MTBC seen by the PHE laboratory in Birmingham, we applied the algorithm to 3,128 MTBC genome sequences from consecutively obtained clinical isolates. H37Rv is routinely sequenced by the laboratory on WGS plates; these isolates were not removed, and their identification served as an internal control.

### Comparison with Existing Tools

We first compared strain characterization by our new SNP-IT tool with those of KvarQ (https://github.com/kvarq/kvarq), TB-Profiler (http://tbdr.lshtm.ac.uk), and Mykrobe predictor (http://www.mykrobe.com/products/predictor) on default settings and then after integrating our updated SNP library. To enable our new data to be integrated with published SNP libraries (*23*) and for practical reasons when modifying existing tools, we created a minimal SNP dataset. We filtered our larger SNP catalog for synonymous SNPs that occurred in coding regions (as annotated by SnpEff version 4.3 [*24*]) and selected 1 representative SNP for each subspecies, lineage, and sublineage at random. We then modified the existing software packages to include reference SNPs (or kmers for Mykrobe predictor) for the subspecies, lineages, or sublineages that they initially failed to identify.

## Results

### Calibration and Validation

**Figure 2:**
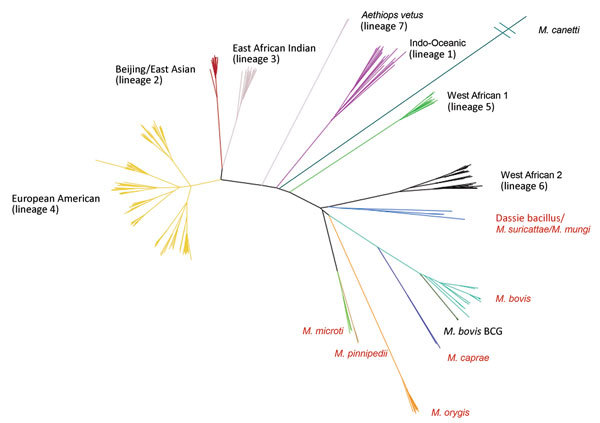
Maximum-likelihood tree built from 70,144 informative positions from whole-genome sequences of all 323 *Mycobacterium tuberculosis* complex samples in the calibration set for the new SNPs to Identify TB tool. Lineages are of *Mycobacterium tuberculosis*. Red text denotes animal subspecies. BCG, bacillus Calmette–Guérin; SNP, single-nucleotide polymorphism.

In total, we identified 13,893 SNPs (median of 229 SNPs per group, interquartile range 296) as predictive of taxonomic and phylogenetic groups of interest (Appendix Table 3). The greatest number of phylogenetic SNPs was seen in *M. canettii* (n = 6,837) and the fewest in *M. bovis* (n = 23). Subspecies that arise from common deep branches, such as *M. microti* and *M. pinnipedii* (Figure 2), have lower numbers of unique phylogenetic SNPs (n = 128 for *M. microti* and n = 301 for *M. pinnipedii*) than those that do not, such as *M. orygis* (n = 781). All predictions made by SNP-IT across all the subspecies, lineages, and sublineages were consistent with the maximum-likelihood phylogeny for all isolates in the validation set (Table 1).

**Table 1:** Comparison between speciation calls for 614 MTBC samples in validation set made by SNP-IT and position on maximum-likelihood phylogenetic tree*

Species (lineage)	SNP-IT typing calls	Maximum-likelihood calls	% Correct
*Mycobacterium bovis* BCG	22	22	100%
*M. bovis*	15	15	100%
*M. orygis*†	31	31	100%
*M. microti*†	18	18	100%
*M. canettii*†	34	34	100%
*M. pinnipedii*†	8	8	100%
*M. caprae*†	15	15	100%
Dassie bacillus†	2	2	100%
*M. suricattae*†	2	2	100%
*M. tuberculosis*			
Indo Oceanic (lineage 1)	44	44	100%
Beijing/East Asian (lineage 2)	42	42	100%
East African Indian (lineage 3)	46	46	100%
European American (lineage 4)‡	18	18	100%
Lineage 4 sublineages			
Ghana (4.1)	12	12	100%
X-type (4.1.1)	45	45	100%
Haarlem (4.1.2.1)	45	45	100%
Ural (4.2.1)	19	19	100%
Tur (4.2.2.1)	25	25	100%
LAM (4.3)	37	37	100%
S-type (4.4.1.1)	45	45	100%
Uganda (4.6.1)	18	18	100%
Cameroon (4.6.2.2)	32	32	100%
West African 1 (lineage 5)	13	13	100%
West African 2 (lineage 6)	11	11	100%
*M. aethiops vetus* ( lineage 7)	15	15	100%

*Numbers in parentheses represent the lineage numbering scheme of Coll for the major lineages 1–7 and Stucki for the lineage 4 sublineages. BCG, bacillus Calmette–Guérin; MTBC, *Mycobacterium tuberculosis* complex; SNP, single-nucleotide polymorphism; SNP-IT, SNPs to Identify TB. †Validation set for subspecies augmented with published strains*.*  ‡No sublineage call made.

### Determining Prevalence of Animal Subspecies in a Collection of Clinical Isolates

We retrospectively applied SNP-IT to clinical isolates sequenced as part of the routine PHE diagnostic workflow in Birmingham to estimate the prevalence of the animal subspecies among MTBC samples. Of 3,128 samples from 2,106 patients for which there was a whole-genome sequence available, we identified 24 as *M. orygis*, 3 as *M. microti*, 34 as *M. bovis*, and 1 as *M. caprae* (Table 2). In the case of *M. orygis*, we further investigated whether there was any genomic signal of possible person-to-person transmission. We identified 2 such instances, 1 in which the pairwise genetic distance between 2 patients was 0 SNPs and a second in which it was 6 SNPs (Appendix Figure 1).

**Table 2:** Speciation predictions for collection of 3,128 clinical MTBC isolates from 2,106 patients using SNP-IT*

Species and subspecies (lineage)	No. isolates (no. patients)
*Mycobacterium tuberculosis*	
Indo-Oceanic (lineage 1)	240 (208)
Beijing/East Asian (lineage 2)	242 (175)
East African Indian (lineage 3)	775 (644)
European American (lineage 4); no sublineage call made†	512 (+ 368 H37Rv‡) (336)
Lineage 4 sublineages	
Ghana (4.1)	5 (4)
X-type (4.1.1)	197 (159)
Haarlem (4.1.2.1)	266 (213)
Ural (4.2.1)	43 (34)
Tur (4.2.2.1)	41 (36)
LAM (4.3)	213 (159)
S-type (4.4.1.1)	60 (45)
Uganda (4.6.1)	13 (12)
Cameroon (4.6.2.2)	40 (32)
West African 1 (lineage 5)	4 (2)
West African 2 (lineage 6)	11 (9)
No call made	10 (10)
*M. bovis *BCG	26 (20)
*M. bovis*	34 (28)
*M. orygis*	24 (19)
*M. microti*	3 (2)
*M. caprae*	1 (1)
Total	3,128 (2,106)

*Numbers in parentheses represent the lineage numbering scheme of Coll for the major lineages 1–7 and Stucki for the lineage 4 sublineages. BCG, bacillus Calmette-Guérin; MTBC, *Mycobacterium tuberculosis* complex; SNP, single-nucleotide polymorphism; SNP-IT, SNPs to Identify TB. †These samples belong to lineage 4 but not to one of the named sublineages. ‡SNP-IT correctly identified 880 isolates as being lineage 4; however, 368 of these were identified as being H37Rv when we unblinded ourselves to their laboratory records.

### Phylogenetic SNPs in Drug Resistance–Associated Genes

Using a previously published list of drug resistance–associated genes for *M. tuberculosis* (*25*), we searched all subspecies for phylogenetic SNPs in drug resistance–associated genes (Appendix Table 4). All subspecies contain unique phylogenetic SNPs (N = 95 in total) in these genomic regions, but on the basis of our data, we were unable to determine whether any of these mutations are linked to lineage-specific resistance because we did not have the corresponding phenotypic drug susceptibility testing data.

### Comparison with Existing Software

Compared with SNP-IT for the clinical set of isolates, Mykrobe predictor reported *M. orygis* as *M. tuberculosis* West African lineage and *M. pinnipedii* as *M. microti*. KvarQ identified all animal-associated subspecies only as “animal lineage.” TB-Profiler was unable to delineate among animal subspecies, which were all reported as *M. bovis/M. tuberculosis* West African lineage.

After we modified the KvarQ, TB-Profiler, and Mykrobe predictor databases with our minimal SNP catalog, all systems agreed on the identity of all the MTBC isolates in the clinical set. SNP-IT was unable to identify 10 samples because <10% of type-specific SNPs were present in these strains. This result was because our pipeline made no call at the lineage informative sites because of the presence of a minor allele, most likely the result of contamination or a mixture of 2 strains in the sample. However, TB-Profiler and Mykrobe predictor were both able to identify 2 of these isolates as mixed Beijing lineage/*M. orygis* using our new minimal SNP dataset.

## Discussion

SNP typing is a powerful method for discriminating among members of MTBC, which are often not discernible by conventional laboratory methods. The SNP databases of Stucki, Coll, and Comas are currently used as the knowledge base for KvarQ, TB-Profiler, and Mykrobe predictor (*18*,*23*,*26*). None of these databases, however, provides adequate resolution for the animal subspecies. In contrast, SNP-IT was able to assign subspecies, lineages, and sublineages to all samples in the validation set with 100% accuracy compared with maximum-likelihood phylogeny. Implementing this fine-resolution algorithm into a routine diagnostic workflow would be a major step toward understanding the epidemiology and pathogenicity of the less common members of MTBC. All 3 existing systems tested (KvarQ, Mykrobe predictor, and TB-Profiler) were identical in performance when given the same SNP reference database, demonstrating that the clinically meaningful differences highlighted in a recent review are easily ameliorated (*27*).

By applying SNP-IT to a clinical dataset, we discovered an unexpectedly high number of animal subspecies among MTBC isolates, particularly *M. orygis*, from humans in the United Kingdom. This recently described member of the complex has a host range that includes waterbucks, gazelles, rhesus monkeys, cows, and rhinoceri (*2*,*28*,*29*). Several human cases have been described in patients in the Netherlands of South/Southeast Asian origin (*2*), but no cases have been described in the United Kingdom. Human-to-animal transmission has been described in 1 case in New Zealand (*30*). Given that zoonotic TB is associated with higher rates of extrapulmonary disease and may be less likely to grow in culture (*31*,*32*), retrospective interrogation of WGS libraries, as in this study, is likely to underestimate the true burden of disease.

Given the large amount of resources aimed at controlling bovine TB, it is noteworthy that another zoonosis is seen at a similar rate in this collection of clinical isolates. This finding raises questions about the host range and transmission of *M. orygis*, with potential implications for TB control both in animals and humans. To recognize the particulars of the clinical phenotype, epidemiology, and optimal management strategy of *M. orygis* infection, it is first crucial to accurately distinguish these cases from *M. tuberculosis* West African lineages (*5**,**6*). This discernment is currently not possible by either the Hain Genotype MTBC molecular probe or existing SNP-based platforms. We identified 2 pairs of nearly identical *M. orygis* isolates that could be compatible with either person-to-person transmission or, possibly, common exposure to the same infected animal. From an international perspective, the role of *M. orygis* in zoonotic transmission in Africa, Asia, and other high-prevalence settings with extensive animal contact is poorly understood and may warrant further investigation.

All the animal subspecies had phylogenetic SNPs in drug resistance–associated genes. When these genes are not known to be associated with drug resistance, they can be helpfully annotated as such by diagnostic algorithms and excluded for the purpose of predicting susceptibility. An unavoidable weakness of any SNP-based approach is its vulnerability to null-calls as a result of minor alleles at informative positions or a lack of coverage. SNP-IT uses the entire library of subspecies/lineage/sublineage-defining SNPs such that this weakness is not an issue unless it occurs at most of these positions. An additional limitation is that, although we have sought to calibrate SNP-IT using the most diverse collection of samples available to us, it may not be able to correctly identify isolates that originate from deeper phylogenetic branches than those in our calibration set.

In conclusion, in this study we demonstrate a higher-than-expected burden of zoonotic TB in a large collection of clinical isolates from the United Kingdom. The SNP-IT tool we have developed will help researchers to examine the epidemiology of zoonotic TB in a global context, as well as optimizing the disease’s clinical management. As more healthcare systems begin to routinely use WGS, there is an opportunity to accurately diagnose the causative subspecies of TB in all cases, which will enable identification of previously underrecognized zoonoses and reverse zoonoses and implementation of control interventions in the interests of One Health.

Appendix Additional data about subspecies and associated lineages of *Mycobacterium tuberculosis* complex.
