# Supplementary material for: SNP-IT Tool for Identifying Subspecies and Associated Lineages of Mycobacterium tuberculosis Complex
Source: Emerg Infect Dis. 2019 Mar;25(3):482–8. doi: 10.3201/eid2503.180894 (PMC6390766; doi:10.3201/eid2503.180894)
Supplement: Appendix — Additional data about subspecies and associated lineages of Mycobacterium tuberculosis complex. [file 18-0894-Techapp-s1.pdf]

Article DOI: https://doi.org/10.3201/eid2503.180894

SNP-IT Tool for Identifying Subspecies and Associated Lineages of *Mycobacterium tuberculosis* Complex

Appendix

Post Hoc Analysis of Lineage 4 Genomes

Within the validation set we included 18 genomes from lineage 4 for which no further sublineage could be assigned by SNP-IT and which we therefore defined by the presence of conserved bases relative to the reference. Their position on a maximum likelihood tree revealed that these were phylogenetically distinct from other currently named sublineages of lineage 4. We therefore carried out a post hoc analysis of all genomes in the validation and clinical sets labeled by SNP-IT as being "lineage 4" with no further sublineage name given, comparing their position on the maximum likelihood tree to the nomenclature of Coll (*1*) and Stucki (*2*) (Appendix Figure 1). Of 886 genomes labeled "lineage 4" with no further sublineage identification by SNP-IT, most were lineage 4.10 (N = 817) (Stucki), also known as lineage 4.7 (N = 35), 4.8 (N = 247), and 4.9 (N = 535) (Coll). A minority belonged to lineage 4.5 (N = 35) and lineage 4.6 (N = 27) where they were phylogenetically distinct from the Uganda and Cameroon sublineages. The remainder (N = 7) were not further classifiable from lineage 4 by Coll nomenclature. We subsequently updated our SNP catalog for lineage 4 to include these unnamed sublineages using the same methods as we described for the original calibration set. Lineage 4.9 had 368 samples which were found to be H37Rv when we unblinded ourselves to their corresponding laboratory records.

References

1. Coll F, McNerney R, Guerra-Assunção JA, Glynn JR, Perdigão J, Viveiros M, et al. A robust SNP barcode for typing *Mycobacterium tuberculosis* complex strains. Nat Commun. 2014;5:4812. http://dx.doi.org/10.1038/ncomms5812

2. Stucki D, Brites D, Jeljeli L, Coscolla M, Liu Q, Trauner A, et al. *Mycobacterium tuberculosis* lineage 4 comprises globally distributed and geographically restricted sublineages. Nat Genet. 2016;48:1535–43. http://dx.doi.org/10.1038/ng.3704

3. Somoskovi A, Dormandy J, Parsons LM, Kaswa M, Goh KS, Rastogi N, et al. Sequencing of the pncA gene in members of the *Mycobacterium tuberculosis* complex has important diagnostic applications: Identification of a species-specific pncA mutation in "*Mycobacterium canettii*" and the reliable and rapid predictor of pyrazinamide resistance. J Clin Microbiol. 2007;45:595–9. http://dx.doi.org/10.1128/JCM.01454-06

4. de Jong BC, Antonio M, Gagneux S. *Mycobacterium africanum*—review of an important cause of human tuberculosis in West Africa. PLoS Negl Trop Dis. 2010;4:e744. http://dx.doi.org/10.1371/journal.pntd.0000744

5. van Ingen J, Rahim Z, Mulder A, Boeree MJ, Simeone R, Brosch R, et al. Characterization of *Mycobacterium orygis* as *M. tuberculosis* complex subspecies. Emerg Infect Dis. 2012;18:653–5. http://dx.doi.org/10.3201/eid1804.110888

6. Bruker-Hain Diagnostics. Hain genotype Mtbc [cited 2017 Jan 1]. https://www.hain-lifescience.de/en/products/microbiology/mycobacteria/tuberculosis/genotype-mtbc.html

7. Ross BC, Raios K, Jackson K, Dwyer B. Molecular cloning of a highly repeated DNA element from *Mycobacterium tuberculosis* and its use as an epidemiological tool. J Clin Microbiol. 1992;30:942–6.

8. van Soolingen D, de Haas PE, Hermans PW, Groenen PM, van Embden JD. Comparison of various repetitive DNA elements as genetic markers for strain differentiation and epidemiology of *Mycobacterium tuberculosis*. J Clin Microbiol. 1993;31:1987–95.

9. van Soolingen D, de Haas PEW, Hermans PWM, van Embden JDA. DNA Fingerprinting of *Mycobacterium tuberculosis*. In: Methods in Enzymology. Abelson J, Simon M, Verdine G, Pyle A, editors. New York: Academic Press; 1994. p. 196–205.

10. Van Embden JD, Cave MD, Crawford JT. Strain identification of *Mycobacterium tuberculosis* by DNA fingerprinting: recommendations for a standardized methodology. J Clin Microbiol. 1993;31:406–9. http://jcm.asm.org/content/31/2/406.short

11. Kamerbeek J, Schouls L, Kolk A, van Agterveld M, van Soolingen D, Kuijper S, et al. Simultaneous detection and strain differentiation of *Mycobacterium tuberculosis* for diagnosis and epidemiology. J Clin Microbiol. 1997;35:907–14.

12. Supply P, Allix C, Lesjean S, Cardoso-Oelemann M, Rüsch-Gerdes S, Willery E, et al. Proposal for standardization of optimized mycobacterial interspersed repetitive unit-variable-number tandem repeat typing of *Mycobacterium tuberculosis*. J Clin Microbiol. 2006;44:4498–510. http://dx.doi.org/10.1128/JCM.01392-06

13. de Beer JL, Akkerman OW, Schürch AC, Mulder A, van der Werf TS, van der Zanden AGM, et al. Optimization of standard in-house 24-locus variable-number tandem-repeat typing for *Mycobacterium tuberculosis* and its direct application to clinical material. J Clin Microbiol. 2014;52:1338–42. http://dx.doi.org/10.1128/JCM.03436-13

14. Comas I, Coscolla M, Luo T, Borrell S, Holt KE, Kato-Maeda M, et al. Out-of-Africa migration and Neolithic coexpansion of *Mycobacterium tuberculosis* with modern humans. Nat Genet. 2013;45:1176–82. http://dx.doi.org/10.1038/ng.2744

15. Nebenzahl-Guimaraes H, Yimer SA, Holm-Hansen C, de Beer J, Brosch R, van Soolingen D. Genomic characterization of *Mycobacterium tuberculosis* lineage 7 and a proposed name: ‘Aethiops vetus’. Microb Genom. 2016;2:e000063. http://dx/doi.org/10.1099/mgen.0.000063

Appendix Table 1. Identification methods for training isolates acquired from the Netherlands National Institute for Public Health and the Environment (RIVM)*

Sample ID	Species	Human/Animal	Sex	Age at diagnosis	Date of isolation	Country of origin	Method of identification
7da4f935-2b2f-4640-9fd6-ea4dac70ef43	*M. microti*	Animal (mouse)	Unknown	Unknown	Unknown	UK	VNTR, spoligo, RFLP, PGRS
a5706cea-2eeb-4dc0-ae64-47e4d6d5fbb3	*M. microti*	Human	Male	57	2002 May 24	NL	VNTR, RFLP
b1fa7231-fa7a-4ba9-afbd-eb2b96444118	*M. microti*	Human	Female	59	2015 Oct 8	NL	VNTR, Hain MTBC
f206f3a5-d127-4698-a0ec-19a524c408ac	*M. canettii*	Human	Unknown	Unknown	1993 Feb 10	NL	VNTR, spoligo, RFLP, pncA (*3*)
46a56d43-ac15-48fd-81b5-846e12bc6d10	*M. canettii*	Human	Male	34	2002 Jun 10	NL	VNTR, RFLP, pncA (3)
fbd67644-f009-4f78-9f14–5a21683d30d5	*M. canettii*	Human	Male	25	2007 Oct 19	NL	VNTR, spoligo, RFLP, pncA (*3*)
7ea3d4d6-a416-49bb-8c01-edf4efbd2cd8	*Mtb* West African 1 (lineage 5)	Human	Unknown	Unknown	1995 Oct 18	NL	VNTR, RFLP
8307cd56-a417-4f33–8c65–335ba11cc0c5	*Mtb* West African 2 (lineage 6)	Human	Unknown	Unknown	1993	NL	VNTR, molecular classification (*4*)
6a63a9bb-6eef-4baa-8be1-b06bb0e31b97	*Mtb* West African 2 (lineage 6)	Human	Unknown	Unknown	1993	NL	VNTR, molecular classification (*4*)
25c63622-317b-4387-8484-da79bf7543a6	*M. pinnipedii*	Animal	Unknown	Unknown	1992–93	Argentina	VNTR, spoligo, RFLP, PGRS
f0d95ba8-52bf-46eb-a196-c60147186e84	*M. pinnipedii*	Animal	Unknown	Unknown	1992–93	Argentina	VNTR, spoligo, RFLP, PGRS
df23492d-6f79-475e-8654-a4256e79c5d4	*M. pinnipedii*	Animal (seal)	Unknown	Unknown	2006	NL	VNTR, spoligo
2fec2a96-cc9a-4895-a01f-24a6c30d4235	*M. caprae*	Human	Male	52	2006 Nov 30	NL	VNTR, Hain MTBC
ee2503c3–52aa-4b64-a5ba-eff58036a05a	*M. caprae*	Human	Female	22	2006 Dec 15	NL	VNTR, Hain MTBC

Sample ID	Species	Human/Animal	Sex	Age at diagnosis	Date of isolation	Country of origin	Method of identification
ed1e9f45-ace3-464d-b48e-b5038a68b227	*M. aethiops vetus* (Mtb lineage 7)	Human	Unknown	Unknown	Unknown	Ethiopia	VNTR
26af0867-691e-41da-aa79-3e531895ec33	*M. aethiops vetus* (Mtb lineage 7)	Human	Unknown	Unknown	Unknown	Ethiopia	VNTR
dbc3bd41-21a6-4164-b4d5-c1c9900d6e8a	*M. orygis*	Human	Male	42	2013 Mar 20	NL	VNTR, pncA (*5*)
e2f10303-774d-4b68-b3fa-d2b00f5eded0	*M. orygis*	Human	Female	81	2013 Jun 19	NL	VNTR, pncA (*5*)
c2140309-094d-46e0-8d18-8689c0475e38	*M. bovis BCG*	Human (vaccination)	Male	7	2015 Dec 10	NL	VNTR, Hain MTBC
c64bc667-ac80-4a90-9a40-685c13e542fb	*M. bovis BCG*	Human (bladder carcinoma)	Male	69	2016 May 19	NL	VNTR, Hain MTBC

*BCG, bacillus Calmette-Guerin; Hain MTBC, Hain Genotype MTBC line probe assay (*6*); NL, Netherlands; PGRS, polymorphic GC-rich repeat sequence (*7,8*); RFLP, restriction fragment length polymorphism (*9,10*); spoligo, spacer oligonucleotide typing (*11*); VNTR, variable number tandem repeat (*12,13*).

Appendix Table 2. Nomenclature for the *Mycobacterium tuberculosis* complex members adopted in this study; *Mtb* lineage numbering shown according to the system proposed by Comas/Gagneux (*14*).

Subspecies	Lineage
M. tuberculosis	Indo-Oceanic (*Mtb* lineage 1)
	Beijing/East Asian (*Mtb* lineage 2)
	East African Indian (*Mtb* lineage 3)
	European American (*Mtb* lineage 4) sublineages: Haarlem, LAM, Cameroon, Ghana, S-type, Tur, Uganda, Ural, X-type
	West African 1 (*Mtb* lineage 5)
	West African 2 (*Mtb* lineage 6)
	M. aethiops vetus (*Mtb* lineage 7) (*15*)
M. orygis	
M. bovis	
M. caprae	
M. bovis BCG	
M. pinnipedii	
M. suricattae	
M. mungi	
M. canettii	
Dassie bacillus	

Appendix Table 3. Unique phylogenetic single nucleotide polymorphisms (SNPs) per subspecies/lineage/sublineage

Subspecies/lineage/sublineage	Unique phylogenetic SNPs
Dassie bacillus	323
M. bovis	23
M. bovis BCG	223
M. orygis	781
M. microti	128
M. canettii	6837
M. pinipedii	301
M. caprae	294
M. mungi	609
M. suricattae	510
Indo-Oceanic (Mtb lineage 1)	425
Beijing/East Asian (Mtb lineage 2)	304
East African Indian (Mtb lineage 3)	235
European American (Mtb lineage 4*)	163
Haarlem	59

Subspecies/lineage/sublineage	Unique phylogenetic SNPs
LAM	102
Cameroon	172
Ghana	186
Uganda	100
S-type	165
Tur	43
Ural	33
X-type	60
West African 1 (Mtb lineage 5)	657
West African 2 (Mtb lineage 6)	343
M. aethiops vetus (Mtb lineage 7)	817

*Conserved bases with respect to the reference.

AppendixTable 4. Phylogenetic single-nucleotide polymorphisms in drug resistance-associated genes in members of the *Mtbc*; National Center for Biotechnology Information reference genome no. NC000962.2.

Position	Ref	Alt	Gene	Position	Ref	Alt	Gene
M. canettii				*M. bovis*			
1822859	A	G	cydD	9217	A	C	gyrA
4243690	T	C	embA	Dassie bacillus			
4244154	A	C	embA	1822202	G	A	cydD
4244312	T	G	embA	413654	G	T	iniC
4245023	A	G	embA	*M. caprae*			
4247590	A	G	embB	2714317	C	T	eis
4247815	C	T	embB	1673766	C	T	fabG1
4248206	A	G	embB	6307	T	G	gyrB
4240237	G	C	embC	*M. microti*			
1417294	T	C	embR	1823237	C	A	cydD
9377	A	G	gyrA	5671	C	T	gyrB
9716	T	C	gyrA	471630	G	C	ndhA
9740	G	A	gyrA	472502	T	C	ndhA
9776	T	C	gyrA	1473079	G	A	rrs
5452	G	A	gyrB	*M. mungi*			
2156055	G	A	katG	4242177	C	A	embC
3645738	T	C	manB	8134	T	C	gyrA
3645930	A	G	manB	1833589	A	G	rpsA
2101777	G	C	ndh	1918158	G	A	tlyA
471908	T	C	ndhA	*M. orygis*			
471944	T	C	ndhA	2726378	T	A	ahpC
472397	G	A	ndhA	1819985	G	C	cydC
2289104	T	C	pncA	1821891	G	C	cydD
1833554	A	G	rpsA	4244154	A	G	embA
1833568	G	C	rpsA	5516	A	G	gyrB
1834912	A	G	rpsA	6109	G	A	gyrB
M. bovis BCG				6717	T	C	gyrB
4247173	G	A	embB	412484	C	G	iniA
8624	G	T	gyrA	2154707	C	G	katG
2102106	C	G	ndh	1834363	G	A	rpsA
781568	C	T	rpsL	*M. suricattae*			
M. pinnipedii				1820784	C	T	cydC
1674520	C	T	inhA	1472059	T	G	rrs
1473094	T	C	rrs				
Indo-Oceanic Mtb lineage 1				Ural			
4245969	C	T	embA	3646964	C	G	rmlD
4241042	A	G	embC	X-type			
1417019	C	T	embR	4249408	G	A	embB
8452	C	T	gyrA	West African 1 Mtb lineage 5			
6112	G	C	gyrB	4244635	T	C	embA
471666	A	G	ndhA	4245147	C	T	embA
3647591	A	G	rmlD	9566	C	T	gyrA
Beijing/East Asian Mtb lineage 2				2101921	C	T	ndh

Position	Ref	Alt	Gene	Position	Ref	Alt	Gene
4243460	C	T	embA	West African 2 Mtb lineage 6			
1834177	A	C	rpsA	4244379	C	T	embA
East African Indian Mtb lineage 3				4241843	C	A	embC
4242075	G	A	embC	1674434	T	C	inhA
3645524	C	T	manB	760969	C	T	rpoB
762434	T	G	rpoB	761723	A	C	rpoB
European American Mtb lineage 4				*M. aethiops vetus* Mtb lineage 7			
Haarlem				4248073	C	T	embB
760115	C	T	rpoB	4240153	G	A	embC
S-type				1416977	T	C	embR
411371	T	C	iniA	8876	C	T	gyrA
2102990	A	G	ndh	412842	A	G	iniC
Uganda				2102218	G	A	ndh
7539	A	G	gyrA	1834916	A	C	rpsA
412017	C	G	iniA	1918281	A	C	tlyA

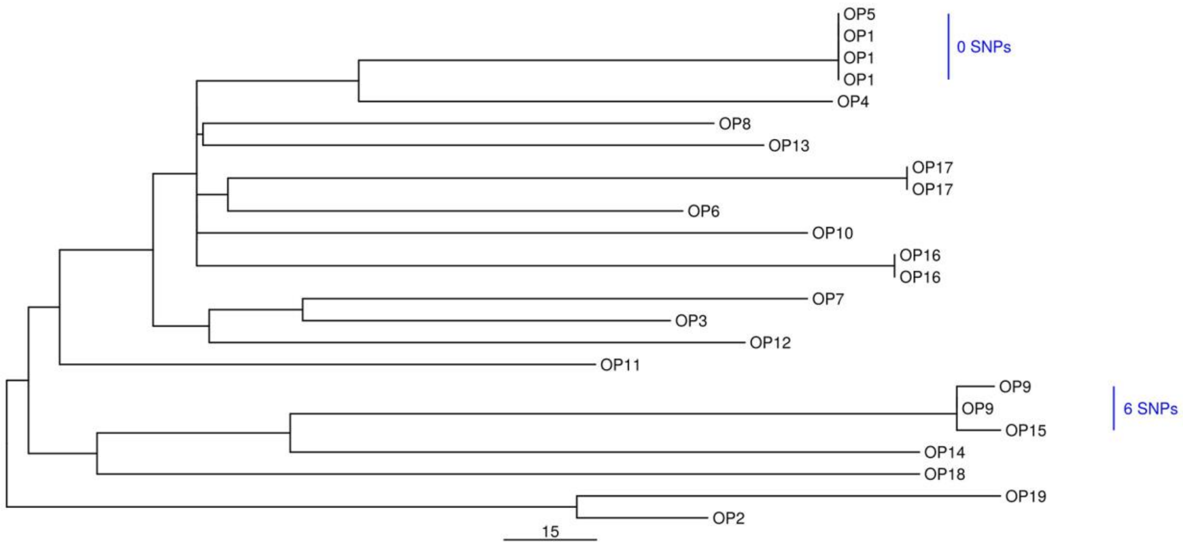

Appendix Figure 1. Maximum likelihood tree of *M. orygis* isolates from the clinical dataset. The 2 instances of patients with a pairwise single-nucleotide polymorphism (SNP) distances that could plausibly support person-to-person transmission or exposure to a common source are highlighted with blue bars. The accompanying SNP distance highlighted in blue is the pairwise distance between the patients. Scale bar indicates nucleotide substitutions per genome. OP, orygis patient.

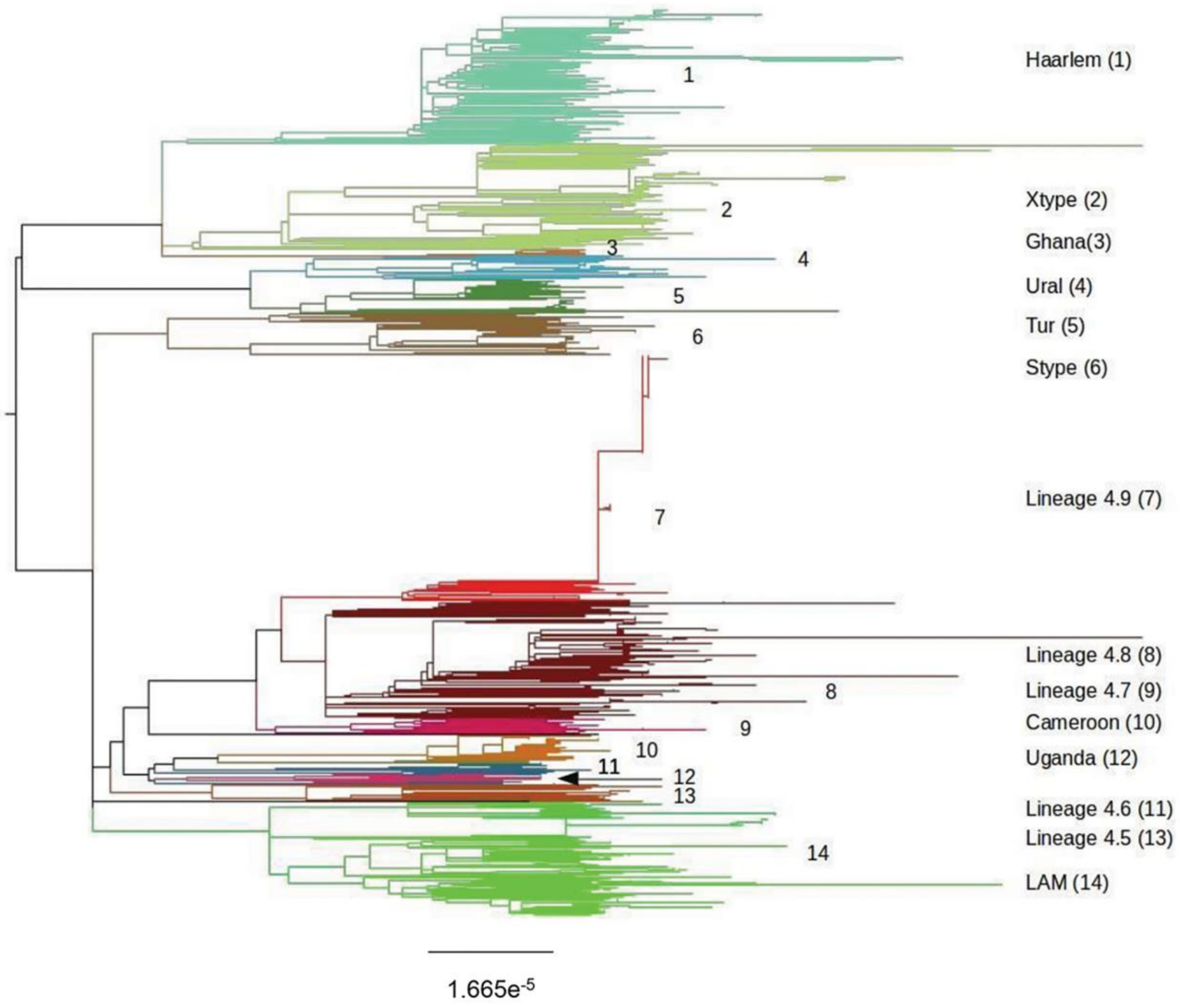

Appendix Figure 2. Maximum likelihood tree of all lineage 4 genomes from the validation and clinical sets. Unnamed lineages are numbered per Coll et al. (*1*). There are a number of lineage 4 sublineages without a name, which are called simply “Lineage 4” in our validation set. We subsequently updated the SNP-IT database to include resolution of all major sublineages as derived by principal components analysis by Stucki et al. (*2*). Scale bar shows nucleotide substitutions per site.
